# Why do patients get idiopathic pulmonary fibrosis? Current concepts in the pathogenesis of pulmonary fibrosis

**DOI:** 10.1186/s12916-015-0412-6

**Published:** 2015-09-24

**Authors:** Pierre-Simon Bellaye, Martin Kolb

**Affiliations:** Firestone Institute for Respiratory Health, St. Joseph’s Healthcare and Department of Medicine, McMaster University Hamilton, Hamilton, Canada; Department of Pathology and Molecular Medicine, McMaster University Hamilton, Hamilton, Canada

**Keywords:** Extracellular matrix, Fibrogenesis, Idiopathic pulmonary fibrosis, Lung injury, Myofibroblast, Wound healing

## Abstract

Idiopathic pulmonary fibrosis (IPF) is a devastating lung disease of unknown origin. Recent findings suggest that IPF results from multiple factors that eventually lead to interstitial lung injury. In the pathogenesis it is likely that complex relationships between genetic predispositions, environmental exposures, and lung infections promote the fibrotic processes causing IPF; it is this complexity and the multiplicity of causes that make the population and clinical course of IPF so heterogeneous. Thus, it is clear that one common factor driving IPF pathogenesis in all patients would be far too simplified of an understanding. In recent years, efforts have been made in finding therapeutic strategies that target disease progression rather than disease onset. The biochemical composition and abnormal stiffness of the matrix might be crucial in controlling the cellular phenotype in fibrotic lungs that promotes disease progression and persistence. Though there has been substantial progress in the IPF field in recent years, much more work is required in order to improve the prognosis associated with this disease.

## Background

Idiopathic pulmonary fibrosis (IPF) is one of the most common diseases classified as a rare disease. The prognosis of IPF is poor, with most patients succumbing to their illness at a rate comparable to aggressive cancers [1]. The causes of IPF remain elusive and are not easy to identify as patients diagnosed are typically at an advanced stage of the disease. Several associated risk factors without a clear causative role have been reported [2], including environmental and occupational exposures, infections, and genetic polymorphisms. To date, there is no ideal therapy for IPF, but at least two drugs have been approved in recent years, both demonstrating a significant impact on disease progression [3, 4]. Understanding the signals involved in the pathogenesis and progression of IPF remain a critical component in discovering new therapies, providing early diagnosis, and preventing disease progression.

### Is IPF an uncontrolled protective process?

Unlike fish or amphibians, evolution has favoured fibrogenesis over regeneration in complex organisms such as mammals [5, 6]. Even if the selective advantage provided by such a “substitution” remains unclear, fibrogenesis certainly benefits survival by preventing blood loss and pathogen invasion through mechanisms of wound closure. The pathological evolution of fibrosis requires the combination of an initial injury, which starts the healing process, and impaired wound healing mechanisms. This view suggests an interaction between environmental and genetic factors in IPF pathogenesis. Several gene mutations have been identified among IPF patients in recent years [7], but whether they are direct cause, predisposition factors, or just associations remains unclear. For instance, surfactant protein and mucin gene mutations can lead to direct epithelial cell injury and death, whereas telomerase gene mutations predispose the epithelium to a pathologic response by favouring an abnormal turnover and repair. However, these mutations only affect 1 % (surfactant), 35 % (mucins), and 3 % (telomeres) of IPF patients, leaving more the 60 % of patients without identified genetic predispositions [8]. Therefore, IPF pathogenesis has to be seen as a process involving several steps in which genetic mutations could represent just one of many important components.

Exposure to inhaled environmental agents, most of all cigarette smoke, represents an important risk factor for IPF. The increased risk of developing IPF remains even after smoke cessation, suggesting the establishment of self-sustained (or autocrine) mechanisms after the initial injury [9, 10]. Cigarette smoke, in addition to epithelial injury, also influences epigenetic changes such as DNA methylation and chromatin modifications that regulate the expression of genes involved in tissue repair and which have an impact on IPF pathogenesis [7]. Infections are common in the IPF population; numerous viruses and bacteria have the potential to cause epithelial cell injury and apoptosis [11] and have the capacity to modulate the host response to injury. In experimental settings, infections seem only able to worsen fibrosis in conjugation with other profibrotic stimuli, suggesting that infections might be co-factors for IPF [12, 13]. In the past decade, the lack of clinical evidence of an ongoing inflammation, as well as the inefficiency of immunosuppressive therapies in IPF, diminished the role of chronic inflammation in IPF pathogenesis [5]. Nevertheless, it cannot be ignored that inflammatory cytokine and immune cell infiltration are found in IPF [14, 15]. We have shown, *in vivo*, that interleukin-1β induces an early inflammation promoting the activation of pro-fibrotic pathways through transforming growth factor (TGF)-β1, able to self-sustain up to day 60 independently of any signs of residual inflammation and trigger clustering of myofibroblasts and collagen similar to myofibroblastic foci observed in humans [16]. This highlights complex relations between the initial injury and the impaired wound healing that might favour the profibrotic processes which lead to IPF.

### The vicious cycle caused by increased lung stiffness

Myofibroblasts are the major producers of the fibrotic extracellular matrix (ECM) which results in the characteristic stiffness of a fibrotic lung, decreased lung volumes, and shortness of breath in patients. *In vitro*, the differentiation of myofibroblasts is strongly correlated with substrate stiffness; it is clear that stiff substrates promote the production of profibrotic mediators and ECM deposition [17, 18], whereas substrates of physiological stiffness inactivate myofibroblasts and favour apoptosis [19]. Activation of Rho Kinase and Focal Adhesion Kinase by increased force tension appears to have a major role in this process and the inhibition of these pathways prevents experimental fibrosis [20–22]. The ECM is a storage of growth factors, such as latent TGF-β1, which are bound to integrins, transmembrane proteins allowing cell-matrix adhesions. An increase in substrate stiffness induces mechanical resistance that favours the release of active TGF-β1 from the integrin promoting myofibroblast activity [23]. These *in vitro* approaches strongly suggest that stiffness alone can drive myofibroblast activation and subsequent ECM deposition. However, they lack the spatial cues that ECM and growth factors encounter in the 3D fibrotic lung *in vivo*. Booth et al*.* [24] showed, very elegantly, that decellularised matrix from IPF but not healthy lungs can drive myofibroblast differentiation and accumulation. Even though the difference in stiffness between non-IPF and IPF lungs was conserved after decellularisation in these studies, it remains unclear whether the altered stiffness alone is responsible for the difference of cell behaviour between the two types of matrix or whether the abnormal composition of the fibrotic ECM also plays a role. Indeed, the “matrisome” of the IPF lung is completely different from the non-IPF lung with many more ECM components as well as more latent TGF-β1 trapped in the matrix [24]. These new techniques are very useful to mimic the native environment of cells, but also have limitations including heterogeneous and non-physiological stiffness of the acellular matrix [25]. Moreover, no study has yet fully described the effect of decellularisation on ECM component preservation and it is possible that proteins trapped in the matrix, which may have a role on cell behaviour, are washed out through the process. For instance, Parker et al*.* [26] demonstrated that the IPF matrix can drive the expression of genes in fibroblasts already highly present in the diseased ECM. This suggests an autocrine feedback loop in which IPF ECM triggers the upregulation of its own abnormal ECM components. This shows that the biochemical composition could be as important as the stiffness of the matrix in controlling the cellular phenotype in fibrotic lungs [26]. Further studies are needed to elucidate the actual contribution of matrix stiffness and composition on myofibroblast differentiation and persistence.

## Conclusions

IPF is a complex disease involving multiple steps which eventually overcome physiological repair mechanisms and lead to fibrosis. Even if the etiologic events causing the onset of IPF remain unknown, decades of research have highlighted the fact that fibrogenesis requires a combination of several factors which cause both epithelial injury and impaired wound healing. It is this complexity and the multiplicity of causes that make the population and clinical course of IPF so heterogeneous. For the time being, it seems more realistic to continue investigating therapeutic strategies that limit disease progression rather that prevent its development. Due to the multiple pathways involved in abnormal fibrogenesis, multi-target therapies appear essential.

## Abbreviations

ECM: Extracellular matrix
IPF: Idiopathic pulmonary fibrosis
TGF-β1: Transforming growth factor-β1
